# RTK/Pseudolite/LAHDE/IMU-PDR Integrated Pedestrian Navigation System for Urban and Indoor Environments

**DOI:** 10.3390/s20061791

**Published:** 2020-03-24

**Authors:** Ruihui Zhu, Yunjia Wang, Hongji Cao, Baoguo Yu, Xingli Gan, Lu Huang, Heng Zhang, Shuang Li, Haonan Jia, Jianqiang Chen

**Affiliations:** 1 Key Laboratory of Land Environment and Disaster Monitoring, MNR, China University of Mining and Technology, Xuzhou 221116, China; zhulucking@163.com (R.Z.); hjcao@cumt.edu.cn (H.C.); 2 State Key Laboratory of Satellite Navigation System and Equipment Technology, 589 ZhongShan Street, Qiaoxi District, Shijiazhuang 050081, China; yubg@sina.cn (B.Y.); ganxingli@163.com (X.G.); hlcetc54@163.com (L.H.); 13582161539@163.com (H.Z.); lishuangcetc54@163.com (S.L.); jiahaonan1022@163.com (H.J.); a243945274@163.com (J.C.)

**Keywords:** RTK, pseudolite, landmarks, carrier phase difference, IMU-PDR, urban and indoor navigation

## Abstract

This paper presents an evaluation of real-time kinematic (RTK)/Pseudolite/landmarks assistance heuristic drift elimination (LAHDE)/inertial measurement unit-based personal dead reckoning systems (IMU-PDR) integrated pedestrian navigation system for urban and indoor environments. Real-time kinematic (RTK) technique is widely used for high-precision positioning and can provide periodic correction to inertial measurement unit (IMU)-based personal dead reckoning systems (PDR) outdoors. However, indoors, where global positioning system (GPS) signals are not available, RTK fails to achieve high-precision positioning. Pseudolite can provide satellite-like navigation signals for user receivers to achieve positioning in indoor environments. However, there are some problems in pseudolite positioning field, such as complex multipath effect in indoor environments and integer ambiguity of carrier phase. In order to avoid the limitation of these factors, a local search method based on carrier phase difference with the assistance of IMU-PDR is proposed in this paper, which can achieve higher positioning accuracy. Besides, heuristic drift elimination algorithm with the assistance of manmade landmarks (LAHDE) is introduced to eliminate the accumulated error in headings derived by IMU-PDR in indoor corridors. An algorithm verification system was developed to carry out real experiments in a cooperation scene. Results show that, although the proposed pedestrian navigation system has to use human behavior to switch the positioning algorithm according to different scenarios, it is still effective in controlling the IMU-PDR drift error in multiscenarios including outdoor, indoor corridor, and indoor room for different people.

## 1. Introduction 

The pedestrian navigation technologies can be divided into relative positioning and absolute positioning. The inertial navigation system (INS) is commonly used for relative positioning which uses the acceleration and angular rate output by inertial measurement unit (IMU) to integrate to obtain velocity and attitude. INS can provide continuous position without being restricted by external conditions. However, the acceleration and angular rate information both include random noises, which result in integral cumulated errors in velocity and heading [1]. Fortunately, a zero velocity detection update (ZUPT) technique was proposed to aid the inertial navigation system (INS) [2,3,4], which made the fact that when the pedestrian’s foot is in contact with the ground (still phase), the actual velocity of the foot is zero, and when the still phase is detected, the velocity derived by INS is used as the measured error and then fed to extended Kalman filter (EKF) to estimate the velocity error, called as INS-EKF-ZUPT (IEZ). However, the IEZ algorithm is unable to estimate the error in heading because it cannot obtain the heading observations. Although a zero angular rate update algorithm (ZARU) was proposed to eliminate the error in heading [5], which is not constrained by the external environment, it had limited ability to correct heading. Heuristic drift elimination (HDE) algorithm [6] and its variants [7,8] were proposed to eliminate heading errors, which are effective in indoor path with linear features. The absolute positioning system GPS is a medium-range circular orbit satellite navigation system which is commonly used for outdoor positioning, navigation, and timing. GPS outdoor positioning technologies include pseudorange-based and carrier phase-based algorithms. Real-time kinematic (RTK) is a carrier phase difference technology which is constructed based on real-time processing of the carrier phase of two stations and that can provide three-dimensional coordinates of the rover in real time using GPS signals. However, under the urban canyon environment, where the satellite navigation signals are blocked by buildings and trees, RTK will fail to achieve high-precision positioning. Seriously, it will result in inability to locate. Therefore, a single positioning method cannot achieve continuous high-precision positioning. GPS integrated with IMU-personal dead reckoning systems (PDR) can be used to solve this problem. Tuan Li, et al. [9] integrated single-frequency multi-global navigation satellite system (GNSS) RTK with microelectro mechanical systems (MEMS)-IMU tightly coupled for resisting the measurement outliers, which can provide continuous and precision positioning under urban environments. Zun Niu et al. [10] combined RTK with an IMU-PDR algorithm which used the ZUPT algorithm to assist RTK for the sake of improving positioning performance in urban areas. If the GPS signals are available, RTK is used, if the GPS signals are unavailable, IMU-PDR is used. The integrated navigation algorithms mentioned above are all more robust and allow higher positioning accuracy in complex environments than the signal navigation system. However, there still remain challenges because of the unavailability of GPS signals in indoor environments. To solve this problem, external wireless positioning methods are widely studied to achieve indoor positioning. WiFi and Bluetooth are popular for indoor positioning and have been used for communication because WiFi has become an indoor infrastructure and Bluetooth has lower power consumption and low cost. Besides, both of WiFi and Bluetooth are supported by smartphone. Jingxue Bi et al. [11] presented an adaptive weighted k-nearest neighbor (KNN) positioning method based on an omnidirectional fingerprint database (ODFD) and twice affinity propagation clustering. Sukhoon Jung et al. [12] proposed WiFi fingerprint-based approaches following log-distance path loss model for indoor positioning. Devanshi et al. [13] gave a brief review of indoor localization based on Bluetooth technology. Futoshi Naya et al. [14] proposed a Bluetooth-based indoor proximity detection method for nursing context awareness. However, WiFi and Bluetooth have low positioning accuracy, which cannot meet the needs of indoor high-precision positioning. The other wireless absolute positioning technologies which can achieve higher positioning accuracy have been studied by some scholars, such as radio frequency identification (RFID) [15], near field communication (NFC) [16], ultra wideband (UWB) [17,18], and ultrasound [19]. Antonio Ramón Jiménez Ruiz et al. [20] presented a tight Kalman Filter (KF)-based INS/RFID integration. André G. Ferreira et al. [21] proposed a loose-coupled fusion of inertial and UWB. Yuan Xu et al. [22] presented an improved tightly coupled model of foot-mounted IMU and UWB. Yun Zhuang et al. [23] integrated INS and PDR in pedestrian navigation applications. However, the coverage of RF-based localization methods works within short distances. Although UWB is a high-precision indoor positioning technology, it is not supported by most smartphones. If UWB is used as the indoor pointing device, we have to use special devices to achieve positioning, which is difficult to promote among consumer users.

Pseudolite is a ground-based transmitter that can transmit signals similar to GNSS [24], which is supported by the GNSS receiver chip. Therefore, it is meaningful to study indoor positioning based on pseudolite. The pseudolite is composed of a multichannel pseudosatellite host and pseudosatellite antennas. The host modulates multiple navigation signals, and each of navigation signal is transmitted by a pseudolite antenna. The pseudolite deployed in indoor rooms can make up for the shortcomings of GPS signals that cannot reach indoors. However, the multipath effect is more complicated indoors. The integer ambiguity of the carrier phase is difficult to calculate and the pseudolite hardware performance is worse than that of GPS satellites. All of these lead to the low performance of a conventional satellite navigation positioning algorithm. To achieve high-precision positioning, Kenjirou Fujii et al. [25] proposed hyperbolic positioning with antenna arrays and multichannel pseudolite for indoor localization, which can only achieve high-precision within a small range. Lu Huang et al. [26] proposed an innovative fingerprint location algorithm for indoor positioning based on array pseudolite, which includes the offline phase and the online phase and needs to collect indoor fingerprints in advance, taking a lot of manpower, and is difficult to maintain. Xingli Gan et al. [27] presented a Doppler differential positioning technology using the BeiDou system (BDS)/GPS indoor array pseudolite system, which uses the Doppler difference equation and known point initialization (KPI) to determinate the velocity and position of the receiver.

Different from existing works, the RTK/Pseudolite/ landmarks assistance HDE (LAHDE)/IMU-PDR integrated pedestrian navigation system proposed in this paper consists of RTK receiver, pseudolite, manmade landmarks, smartphone, and IMU, which seems unusable in real conditions. However, the GNSS receiver chip in smartphones and the appearance of Android P and the application of BCM47755 chipset make the smartphone Xiaomi Mi 8 possible to use RTK to achieve high-precision positioning. Many domestic and foreign units are engaged in indoor positioning technologies that integrate pseudolite with other technologies, such as 5G [28] or RFID [25], and with the dense deployment of 5G and RFID, indoors will be widely covered by pseudolite signals. The Xiaomi Technology Co., Ltd. has developed an IMU embedded in the sole of the shoe [29], which can be used to achieve pedestrian navigation. There are many manmade parallel and vertical corridors indoors, which can be used to correct the heading derived by an IEZ algorithm. Therefore, only one smartphone and a pair of shoes can implement the proposed system in this paper which is usable in the future. The steps or slope in front of gates (manmade landmarks) or Bluetooth deployed at the corridor entrance can be used to identify whether the pedestrian arrives at an indoor corridor or not, but the scene recognition technologies are not the focus of this study. The main contributions of this paper are summarized below:

- RTK/Pseudolite/LAHDE/IMU-PDR integrated pedestrian navigation system for urban and indoor environments was proposed. IEZ algorithm was used with the IMU mounted on foot to implement IMU-PDR. RTK was integrated with IMU-PDR outdoors. Meanwhile, in indoor rooms, where GPS is unavailable, pseudolite replaces GPS to integrate with IMU-PDR. The HDE algorithm was introduced to eliminate heading errors under indoor corridor environments where GPS and pseudolite are both unavailable.
- A high-precision indoor positioning method based on carrier phase difference of pseudolite was proposed. Firstly, the hyperbolic positioning method was used to obtain a reliable initial position, and then a local search method based on carrier phase difference (LSMBCPD) with the assistance of IEZ (LSMBCPD-IEZ) was introduced to estimate the subsequent positions.
- Based on the proposed system, the real experiments were carried out in a cooperation scene. The pedestrian walking trajectories included urban, indoor corridors, and indoor room.

The organization of the paper is as follows: Methods are given in Section 2. The filter design is presented in Section 3. Section 4 is the field and materials. Section 5 shows the results and discussion. Section 6 is the conclusion and future work.

## 2. Materials and Methods

### 2.1. Zero Velocity Update

The performance of ZUPT highly relies on the still-phase detection accuracy. Many methods of still-phase detection are based on the readings of accelerometers or gyroscopes [30,31,32]. In our earlier paper [33], we studied and analyzed the performance of the adaptive threshold algorithm for different pedestrians and different movement motions. The results showed that the performance differed between different people and the adaptive threshold algorithm outperformed the fixed threshold-based algorithm. In this paper, the adaptive thresholds algorithm was not the focus of our research and the experimenter walked at normal speed through the whole trajectory. A still-phase detection method based on both acceleration and angular rate is given below:

(1) The magnitude of acceleration and angular rate respectively at epoch k are calculated as:

(1) $$\left\{ \begin{array}{l} a_{k}=\sqrt{a_{k,x}^{2}+a_{k,y}^{2}+a_{k,z}^{2}}\\ \omega_{k}=\sqrt{\omega_{k,x}^{2}+\omega_{k,y}^{2}+\omega_{k,z}^{2}} \end{array}\right.$$

where $\left(a_{k,x},a_{k,y},a_{k,z}\right)$ and $\left(\omega_{k,x},\omega_{k,y},\omega_{k,z}\right)$ are the three-axis readings of accelerometer and gyroscope:

(2) $$\left\{ \begin{array}{l} a_{k}^{\prime }=\mathrm{abs}(a_{k}-\mathrm{mean}(a(1:initPeriod/samplePeriod)))\\ \omega_{k}^{\prime }=\mathrm{abs}(\omega_{k}-\mathrm{mean}(\omega (1:initPeriod/samplePeriod))) \end{array}\right.$$

where the *initPeriod* is a period of time (20 s in this paper) since the IMU was powered on and the *samplePeriod* is sampling frequency.

(2) The low-pass (LP) is used to smooth the signals of acceleration and angular rate:

(3) $$\left\{ \begin{array}{l} a_{k}^{\prime \prime }=\mathrm{LP}\_\mathrm{filter}(a_{k}^{\prime })\\ \omega_{k}^{\prime \prime }=\mathrm{LP}\_\mathrm{filter}(\omega_{k}) \end{array}\right.$$

where LP_filter(*x*) is the low-pass filtering function for *x*.

(3) A binary parameter for detecting the gait is given as:

(4) $$GD(k)=(a_{k}^{\prime }<th_{a})\&(\omega_{k}^{\prime }<th_{\omega })$$

where $th_{a}=0.1g$ and $th_{\omega }=10^{{}^{\circ}}$ are the predefined thresholds for acceleration and angular rate, respectively. If GD(k) is true, it is assuming still-phase, or it is swing-phase.

The velocity derived from INS without correction by Extended Kalman Filter (EKF) is used as the measurements:

(5) $$\Delta v_{k}=v_{k}-\left[\begin{array}{ccc} 0 & 0 & 0 \end{array}\right]$$

### 2.2. HDE Algorithm with the Assistance of Manmade Landmarks (LAHDE)

**Figure 1:**
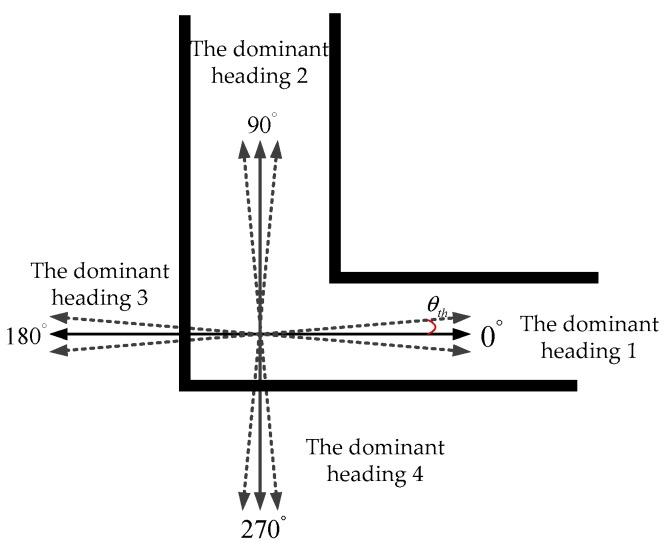
Four dominant directions are pre-established with the fact that most corridors in buildings are straight and so are most walls and sidewalks alongside where a person might walk.

When a pedestrian enters indoor corridor where GPS and pseudolite signals are both unavailable, the positioning results of IEZ algorithm diverge quickly because of the cumulated error in heading. The HDE algorithm and its variants can be seen as landmark-based algorithms, since the manmade straight corridors can be interpreted as landmarks [6]. Figure 1 shows the definition of dominant direction along indoor corridors.

**Figure 2:**
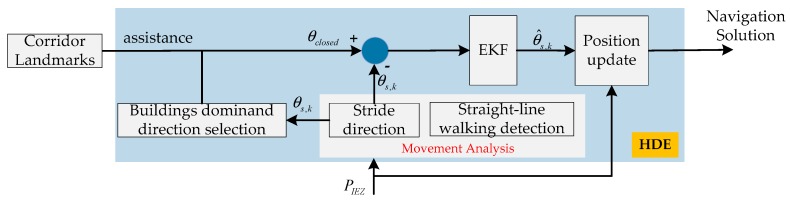
The mechanism of heuristic drift elimination (HDE) algorithm with the assistance of manmade landmarks.

If the HDE algorithm detects that the pedestrian does not move on a straight line, these corrections are suspended. The block diagram of HDE with the assistance of manmade landmarks is shown in Figure 2.

When a pedestrian enters indoor corridors, the HDE algorithm works. The problem is how the pedestrian knows that he arrives at an indoor corridor. This is a scene recognition problem which has to be studied by many scholars using satellite navigation signal, steps, or slope in front of gates (manmade landmarks) and a rough position received by navigation system or Bluetooth deployed at the corridor entrance, and so on. In this paper, however, the scene recognition did not belong to our research content, and we used the pedestrian’s action (press a button) to determine whether the pedestrian entered indoors or not. The detailed description of the HDE algorithm is given in our earlier work [31].

### 2.3. A High-Precision Positioning Method Based on Carrier Phase Difference with the Assistance of IEZ

In this section, the principle of array pseudolite is described firstly. Then, the hyperbolic positioning is introduced to obtain a high-precision initial position. Finally, a local search method based on carrier phase difference with the assistance of IEZ was proposed to achieve the subsequent high-precision positioning.

#### 2.3.1. The Principle of Array Pseudolite

**Figure 3:**
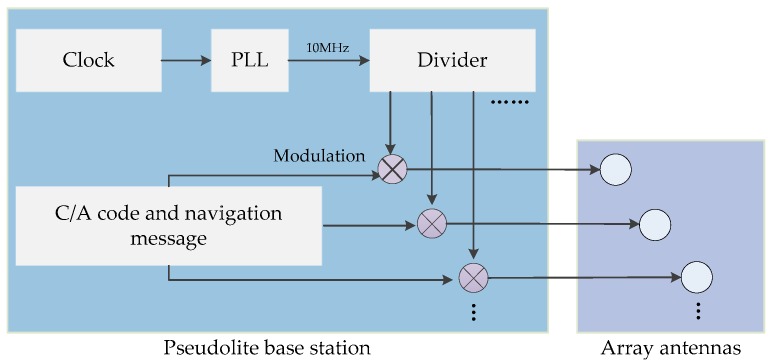
The principle of array pseudolite.

Pseudolite is a ground-based transmitter that can transmit wireless navigation signals similar to GNSS [24], meeting the commercial user receiver chips. The array pseudolite includes a pseudolite host with multiple navigation signal transmission channels and pseudolite array antennas. Each transmission channel was modulated into different C/A codes and navigation messages. Because of the same phase locked loop (PLL) as shown in Figure 3, all channels of array pseudolite simultaneously transmit signals, which can eliminate the clock differences between multiple channels [26].

#### 2.3.2. Hyperbolic Positioning

**Figure 4:**
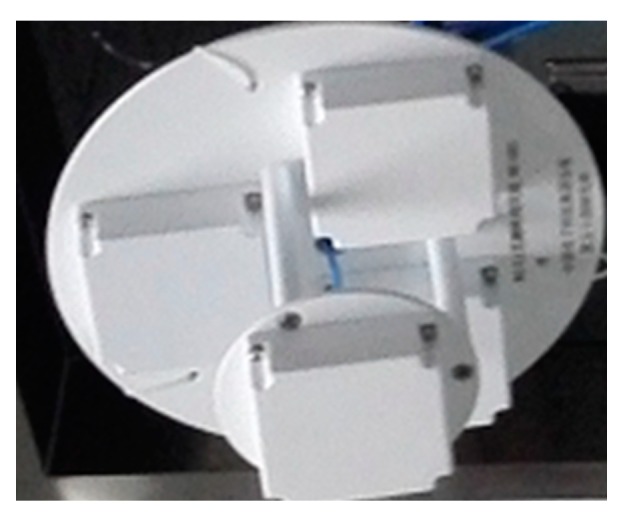
Array antennas.

The hyperbolic positioning using three array antennas can achieve high-precision positioning in a small area, less than 30 cm within a horizontal distance of 0.5 m from the phase center of the array antenna, which is suitable for obtaining an initial high-precision position for the proposed LSMBCPD-IEZ algorithm. Figure 4 shows the antennas’ array consisting of multiple/3 pseudolite antennas, which are deployed at intervals of a half-wavelength of a GPS L1 or BDS B1 carrier wave to each other. The detailed description of the hyperbolic positioning is given in [25].

#### 2.3.3. A Local Search Method Based on Carrier Phase Difference with the Assistance of IEZ (LSMBCPD-IEZ)

In this section, a local search method based on carrier phase difference with the assistance of IEZ was proposed. Firstly, the IEZ was used to predict the stride length of a pedestrian, and then some candidates were generated based on the stride length and a predefined resolution. Finally, an error matching function was introduced to find the optimal candidate. The detailed description of this method is shown below:

Step 1: Stride length. The stride length d was calculated by IEZ algorithm.

**Figure 5:**
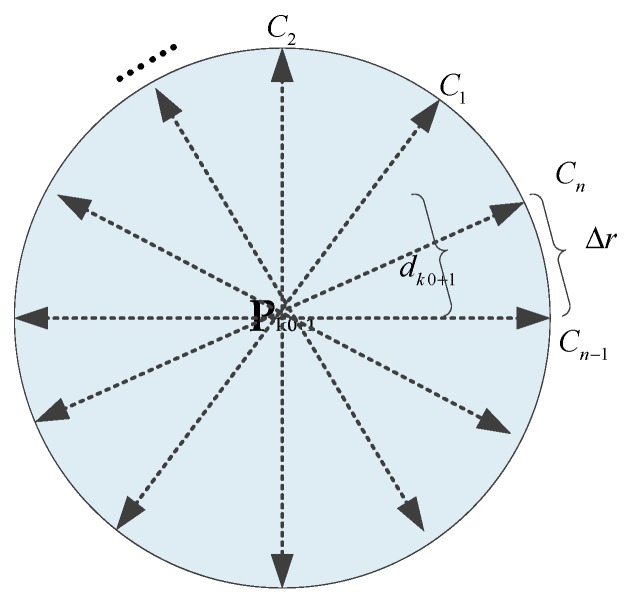
The first candidates, $n=2\pi d_{k0+1}/\Delta r$.

Step 2: First candidates. The initial position was defined as $P_{k0}$, which was derived by the hyperbolic positioning and a circle was drawn with the center $P_{k0}$ and the predicted distance $d_{k0+1}$ at the next sampling epoch *k*0+1. Then, the circle was divided into some discrete points using a predefined resolution, Δr(2 cm), as shown in Figure 5. These discrete points were viewed as the first candidates $\left(C_{1},C_{2},\cdots C_{n}\right)$. Obviously, the positions $\left(P_{1},P_{2},\cdots ,P_{n}\right)$ of all candidates can be calculated by the three parameters $\left(P_{0},d_{k0+1},\Delta r\right)$.

Step 3: The error matching function. The carrier phase observation output by the user receiver was defined as $\varphi_{k0+1}={\left(\varphi_{1},\varphi_{1},\cdots ,\varphi_{n}\right)}_{k0+1}$ at epoch *k*0+1, where *n* is the number of the pseudolite antennas. Taking candidate *C*_1_ as an example, the distance $\Delta d_{k0+1,1}=(\Delta d_{k0+1,(1,1)},\Delta d_{k0+1,(2,1)},\cdots ,\Delta d_{k0+1,(n,1)})$ between *C*_1_ and the antennas was calculated as:

(6) $$\left\{ \begin{array}{l} \left\|P_{pseudolite,1}-P_{1}\right\|=\Delta d_{k0+1,(1,1)}\\ \left\|P_{pseudolite,2}-P_{1}\right\|=\Delta d_{k0+1,(2,1)}\\ \cdots \cdots \\ \left\|P_{pseudolite,n}-P_{1}\right\|=\Delta d_{k0+1,(n,1)} \end{array}\right.$$

where $P_{pseudolite,n}$ is the location of pseudolite antenna *n* and $P_{1}$ is the location of C_1_. The error between the carrier phase observation $\lambda \varphi_{k0+1}$ and $\Delta d_{k0+1,1}$ was calculated as:

(7) $$E_{k0+1,1}=(e_{k0+1,(1,1)},e_{k0+1,(2,1)},\cdots ,e_{k0+1,(n,1)})=\lambda \varphi_{k0+1}-\Delta d_{k0+1,1}$$

The error standard deviation was calculated as:

(8) $$S_{k0+1,1}=\sqrt{\sum_{i}^{n}{\left(e_{k0+1,(i,1)}-\frac{\sum_{j}^{n}e_{k0+1,(j,1)}}{n}\right)}^{2}}$$

Step 1 to Step 3 were repeated to calculate the error standard deviations corresponding to other candidates and the candidate $C_{k}$ with the smallest error standard deviation selected as the result of the local search.

**Figure 6:**
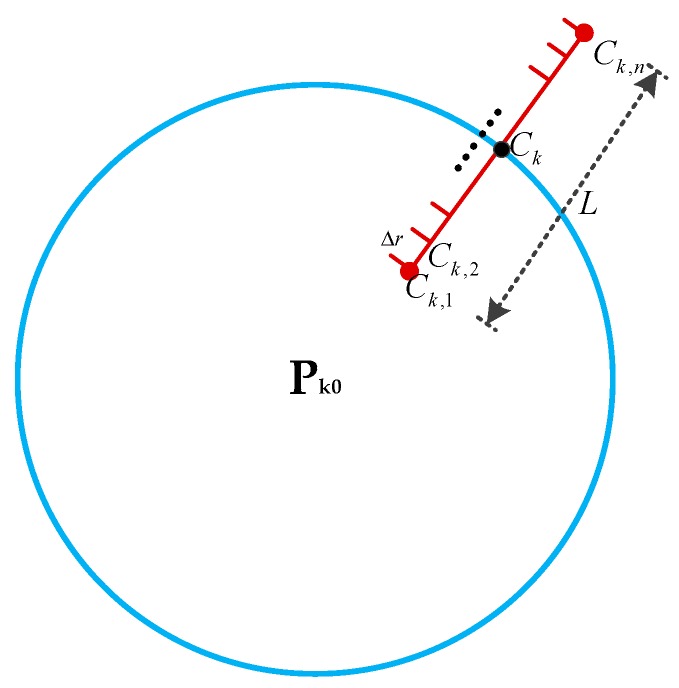
The second candidates, n=L/Δr.

Step 4: The second candidates. Considering that the IEZ algorithm has a relatively small error in stride length, some candidates were generated within the linear range of *L* (40 cm) centered on $C_{k}$ along the $P_{0}C_{k}$ direction as shown in Figure 6.

Step 1 to Step 3 were repeated to find the candidate $C_{k,m}$ responding to the smallest error standard deviation. The position $P_{k,m}$ corresponding to the candidate $C_{k,m}$ was used as the final position estimate $P_{k0+1}$ at epoch *k*0+1. Then, the positions of subsequent sampling epochs were estimated in an indoor room according to the methods of Step 1 to Step 4.

### 2.4. The Brief Description of RTK Theory

In RTK, it mainly depends on the carrier phase and phase correction value sent by the differential reference stations, and the user uses this correction value to perform position calculation, which can achieve centimeter-level accuracy. The key to RTK implementation is how the user receiver obtains high-precision correction value. First, the reference station calculates the carrier phase correction value and transmits it to the rover, and then the rover uses the correction value to correct the observed carrier phase. Finally, the corrected carrier phase observation value is used to form a double difference observer and calculate the rover position.

## 3. Filter Design

**Figure 7:**
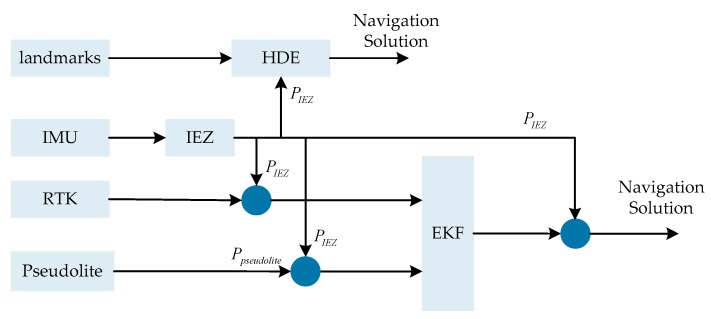
The block diagram of the proposed system.

The EKF was used to fuse the information derived from multisensor outdoor and indoor rooms, and the HDE algorithm with the assistance of landmarks was used to estimate the error in heading derived by IEZ in indoor corridors. The error state model and system measurement model are described as below and the block diagram of the proposed system is shown in Figure 7. 

### 3.1. State Error Model

The error state vector at epoch *k* is

(9) $$\delta x_{k}=[\delta \varphi_{k},\delta \omega_{k},\delta r_{k},\delta v_{k},\delta a_{k}]$$

where $\delta \varphi_{k}$ is the errors in attitude,$\delta \omega_{k}$ is the errors in angular rate,$\delta r_{k}$ is the errors in position,$\delta v_{k}$ is the errors in velocity, and $\delta a_{k}$ is the errors in acceleration. Each of them have three elements. The subscript *k* is the sampling epoch.

The state transition matrix that is a nonlinear function in IMU-PDR navigation is linearized as:

(10) $$\Phi_{k}=\left[\begin{array}{ccccc} I & \Delta t\cdot C_{b,k/k}^{n} & 0 & 0 & 0\\ 0 & I & 0 & 0 & 0\\ 0 & 0 & I & \Delta t\cdot I & 0\\ 0 & 0 & 0 & I & \Delta t\cdot C_{b,k/k}^{n}\\ 0 & 0 & 0 & 0 & I \end{array}\right]$$

The state transition model function is:

(11) $$\delta x_{k+1/k}=\Phi_{k}\delta x_{k}+w_{k}$$

where $\Phi_{k}$ is the state transition matrix,$w_{k}$ is the process noise, and its covariance matrix is $Q_{k}=E(w_{k}w_{k}^{T})$.

### 3.2. Measurement Model

#### 3.2.1. RTK Measurements

Outdoors, where GPS signals are available, RTK is loosely coupled with IEZ for only position solution. The measurement model is as below:

(12) $$P_{RTK}-P_{IEZ}=\Delta P$$

#### 3.2.2. Pseudolite Measurements

In indoor rooms, pseudolite is loosely coupled with IEZ for position solution at the rate of 1 Hz. The measurement model is as below:

(13) $$P_{pseudolite}-P_{IEZ}=\Delta P^{\prime }$$

#### 3.2.3. Velocity Measurements

When the foot was still with the ground, the velocity received from INS without correction by EKF was used as the measurements of the measured error in the velocity:

(14) $$\Delta v_{k}=v_{k}-\left[\begin{array}{ccc} 0 & 0 & 0 \end{array}\right]$$

#### 3.2.4. Heading Measurements

The measured error in heading was calculated as:

(15) $$\Delta \theta_{k}=\theta_{closed}-\theta_{s,k}$$

where $\theta_{closed}$ is the closed dominant direction and $\theta_{s,k}$ is the stride direction calculated by IEZ algorithm at epoch *k*.

## 4. Field and Materials

**Figure 8:**
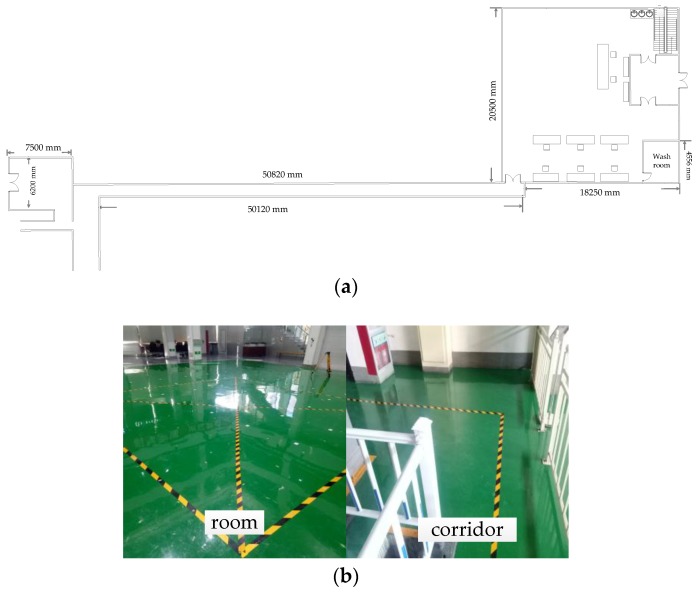
The experiment route of the pedestrian walking. (**a**) The indoor part of the pedestrian walking, (**b**) indoor room and indoor corridors, and the pedestrian walked along yellow-black cross strips.

The real experiments were carried out in Shijiazhuang City, China, to evaluate the performance of the proposed pedestrian navigation system by pedestrian A (a 32-year-old male with a height of 1.78 m and weight of 80 kg) and pedestrian B (a 30-year-old male with a height of 1.80 m and weight of 84 kg). The experimental route of the pedestrian walking was about 600 m and the experimenter walked counterclockwise around the route one loop. The indoor part of the pedestrian walking is shown in Figure 8a. The indoor corridors and room are shown in Figure 8b.

**Figure 9:**
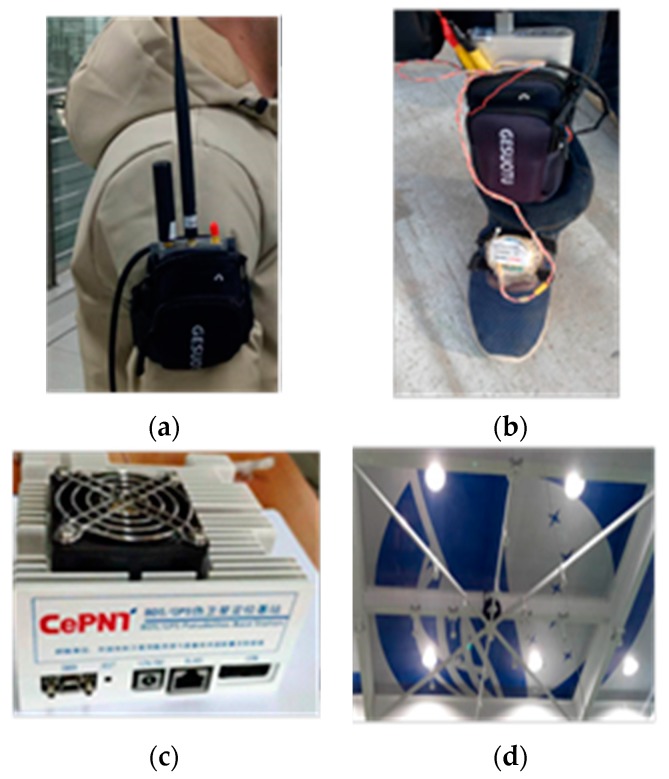
The equipment used in this paper. (**a**) Rover receiver, (**b**) IMU-based pedestrian navigation system, (**c**) pseudolite base station, (**d**) pseudolite antennas.

A mobile application (APP) software was developed to receive and process the data output by the commercial RTK rover and the IMU-based pedestrian navigation system. Figure 9 shows the equipment used in this paper. RTK rover receiver [34] developed by Hexin Xingtong Technology (Beijing) Co., Ltd. Beijing, China, is fixed on the pedestrian’s right arm, shown in Figure 9a, which can simultaneously output global positioning system fix data (GPGGA) data and radio technical commission for maritime services (RTCM) data to smartphone through an external serial to Bluetooth module. The smartphone receives the GPGGA data and then extracts the three-dimensional positions and quality factor using the APP software. The quality factors are shown in Table 1. If it was 4 or 5, the extracted current positions were recorded. An IMU [35] which consisted of three-axis gyroscope and three-axis accelerometer was mounted on the right foot. The IMU-based pedestrian navigation system consisted of an IMU, a microprocessor, and a Bluetooth. The IMU output the raw measurements to the microprocessor at the rate of 100 Hz, and then the microprocessor processed the raw measurements using the IEZ algorithm to estimate the velocity and position of the pedestrian and sent the custom format data, mainly including the estimation positions, to smartphone via Bluetooth low energy (BLE) at each step, as shown in Figure 9b. The smartphone received the custom format data and then extracted the estimated positions. The pseudolite base station is shown in Figure 9c. The antennas were deployed in the indoor room, shown in Figure 9d. When the RTK rover received the pseudolite signals, the APP software parses the RTCM data to extract the carrier phase and then calculated the high-precise position using the LSMBCPD-IEZ method. Filter algorithms were developed using MATLAB to fuse the recorded data using RTK/IMU-PDR, RTK/LAHDE/IMU-PDR, and RTK/Pseudolite/LAHDE/IMU-PDR, respectively.

**Figure 10:**
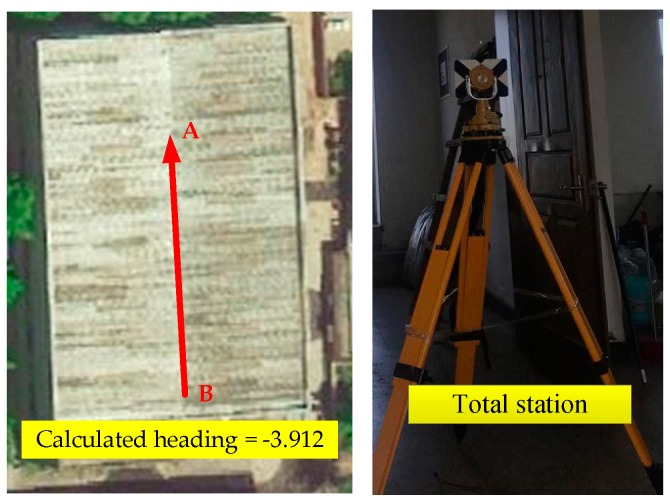
The heading of the corridor.

The Canny method [36] is an effectiveness method for calculating the heading of corridors. In this paper, a total station was used to calibrate the heading of the corridor which was simpler to implement than the Canny method, as shown in Figure 10.

Two points, A and B, from each end of the central axis of the corridor were selected and their positions were calibrated with the total station. The heading of the corridor was calculated as below:

(16) $$\varphi_{south\_gate}=\frac{p_{A,y}-p_{B,y}}{p_{A,x}-p_{B,x}}$$

## 5. Results and Discussion

The real experiment started at a T-junction. The pedestrian travelled from outdoors into N1 building from the south gate, moved along corridors, entered a room where the pseudolite was deployed, then walked counterclockwise along a rectangular path and exited from the north gate of the room, and, finally, returned to the starting point along the outdoor road on the north side of the N1 building. 

### 5.1. The Performance of the Proposed System

**Figure 11:**
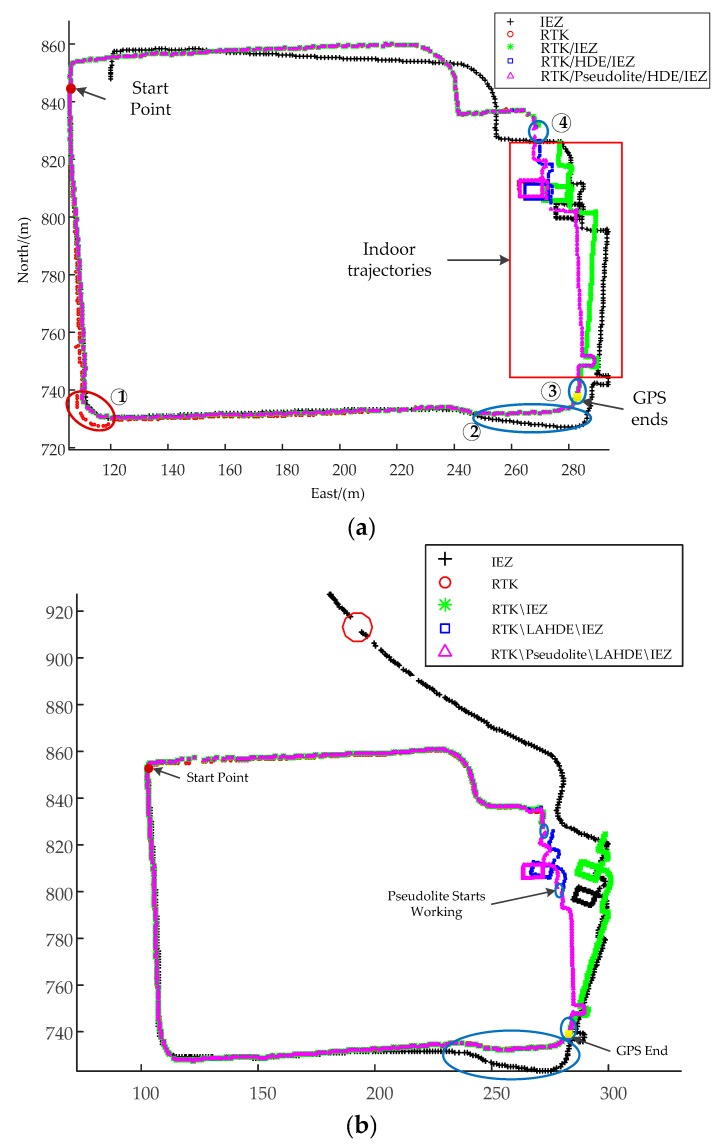
The generated trajectories. (**a**) The trajectories of Person A. (**b**) The trajectories of Person B.

Figure 11 shows the trajectories of Person A and Person B in a Gauss-Kruger (GAUSS) coordinate system with the upper three digits of the *x*-axis and the upper four digits of *y*-axis ignored. As can be seen from Figure 11a, the RTK failed to achieve high-precision in a wooded area environment and the quality indicator was 2, which is about a few meters’ positioning error. The IMU-PDR’s effectiveness for assisting the RTK to improve the system robustness and positioning accuracy is shown in ① in Figure 11a. Because of the accumulated errors in heading, the positioning error of the IEZ algorithm gradually increased, as shown in ② in Figure 11a, and was corrected by RTK. RTK positioning was interrupted when the pedestrian was near the gate of the N1 building because GPS navigation signals were severely obstructed by the N1 building, but the IMU-PDR maintained continuous positioning, as shown in ③ and ④ in Figure 11a, where the scene switching areas were. The walking trajectory of Person B was slight different from that of A. Figure 11b shows the experimental results of Person B, compared with the trajectories of Person A. It can be obviously seen that the performance of the IEZ algorithm differed between Person A and Person B because of the uniqueness of everyone’s motion characteristics which effect the detection accuracy of still phase. It can be obviously seen that the positioning accuracy of the IEZ algorithm of Person A was higher than that of Person B. The performance of adaptive threshold methods were studied and analyzed in our earlier work [31]. The results showed that the adaptive threshold methods outperformed the fixed threshold methods for different people and movement motions. In this paper, the adaptive threshold method was not the focus. Even so, the proposed system had the highest positioning accuracy and robustness than the other coupled solutions and it was able to provide correction to IMU-PDR in a cooperation scene. It can also be seen that the RTK positioning performance of Person B was better than Person A and there was no jump, as shown in ①. This is because Person B walked along the sparse tree area as much as possible. The missing data, as shown in the red circle of Figure 11b, were caused by an interruption in Bluetooth communication.

**Figure 12:**
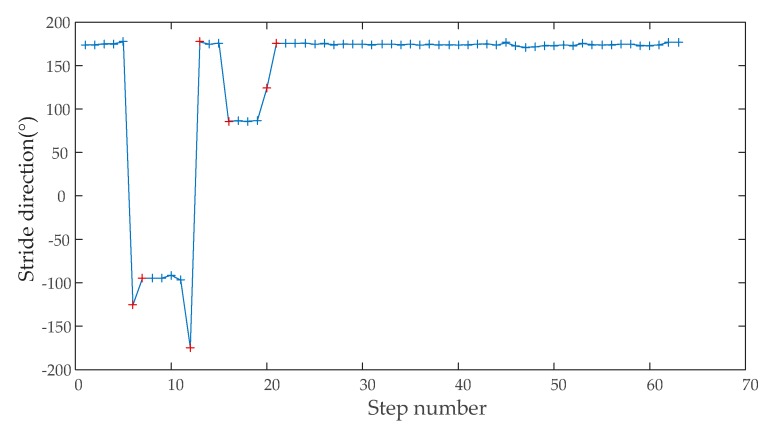
The result of straight walking detection.

When the LAHDE algorithm detected that the pedestrian was walking straight, the stride direction was corrected by the closed dominant direction. Figure 12 shows the strides direction of Person A walking in indoor corridors. The blue-colored ‘+’ represents straight walking and the red ‘+’ represents curved walking. As can be seen, all straight walking paths were detected.

**Figure 13:**
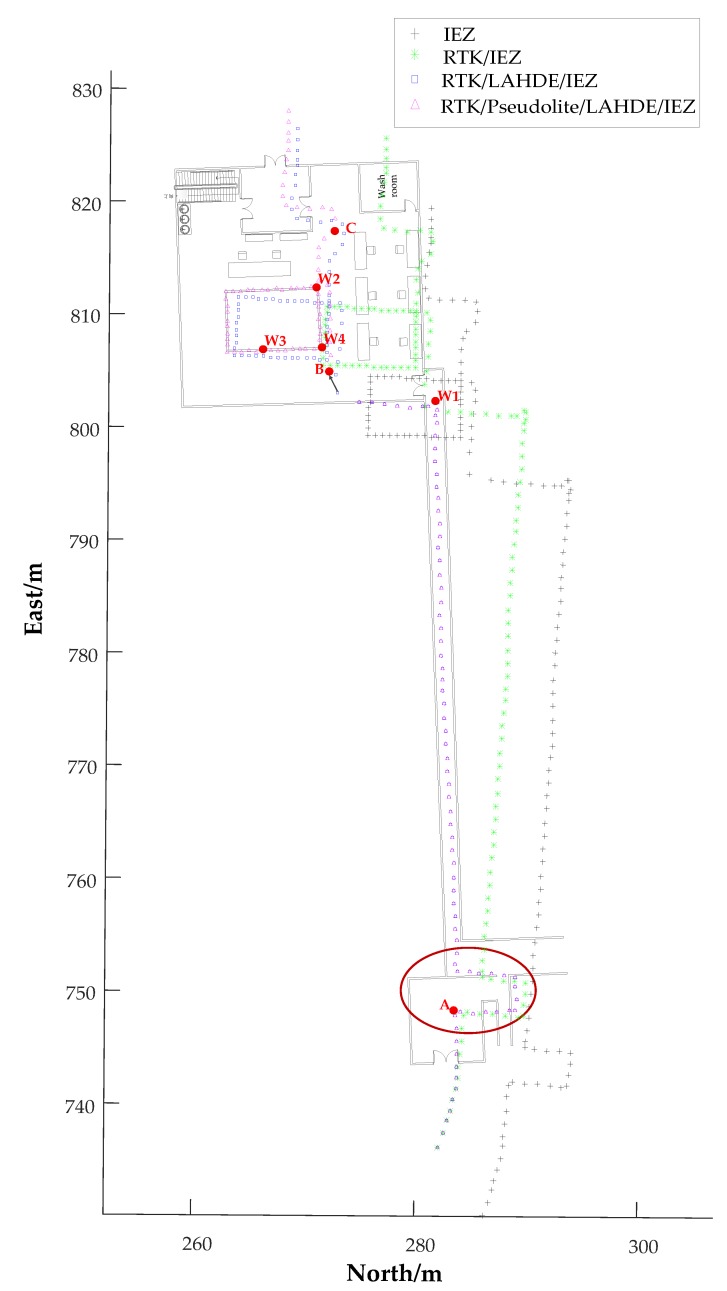
The trajectories of indoor corridor and indoor room parts of Person A.

For better presenting the effectiveness of the proposed system, the trajectories of Person A near and inside N1 buildings were plotted on the map of the building. Seven points were selected as reference locations (waypoints) where the point W1 is the end point of LAHDE algorithm and the point B was the starting point of pseudolite. The trajectories near and inside N1 buildings and the seven waypoints are illustrated in Figure 13. As can be seen, the IEZ algorithm had the worst positioning accuracy. Although the RTK/IEZ can achieve better positioning accuracy than IEZ, it gradually diverged without correction in indoor environments. The RTK/LAHDE/IEZ algorithm had better performance than RTK/IEZ solution and the heading derived by IEZ can be constrained very close to the true value in corridor environment. However, from the red oval as shown in Figure 13, we can see that there was a clear deviation between the pedestrian trajectories and the indoor map. This was because when the pedestrian arrived near the south gate of N1 building, where GPS signals were unavailable, the RTK failed to work. Meanwhile, the HDE algorithm did not start to work because the pedestrian did not arrive at the indoor corridor. The position of point A derived by RTK/LAHDE/IEZ solution was (4212748.35, 538283.65) while the total station calibration result was (4212750.086, 538283.3), with an error of 1.77 m. This phenomenon also occurred at point C. When the pedestrian arrived at point C, the pseudolite signals became seriously unusable due to building occlusion and there was no corridor structure for HDE algorithm implementation. The IEZ algorithm worked as standalone. When the pedestrian arrived at the point B, which was very close to the vertical center of the three-antenna phase center, the point B was corrected to point B` using the hyperbolic positioning method.

We calculated the position errors at the waypoints to evaluate the accuracy of the integrated solutions after GPS ended for IEZ, RTK/IEZ loosely coupled, RTK/LAHDE/IEZ loosely coupled, and RTK/Pseudolite/LAHDE/IEZ loosely coupled systems. The results are shown in Table 2. The position errors grew gradually using IEZ alone. The RTK/IEZ loosely coupled system had better positioning accuracy than IEZ because of the RTK’s correction outdoors. Since RTK cannot work indoors, however, the RTK/IEZ loosely coupled system degenerated into IEZ algorithm and the positioning error started to accumulate in indoor corridors. RTK/LAHDE/IEZ loosely coupled system had better performance than the RTK/IEZ integrated solution because the heading derived by IEZ was constrained very close to the true heading. The RTK/Pseudolite/LAHDE/IEZ loosely coupled system had better performance than the RTK/LAHDE/IEZ integrated solution from point B because the pseudolite started to work from point B and the average of positioning errors using the pseudolite was 0.365 m.

**Figure 14:**
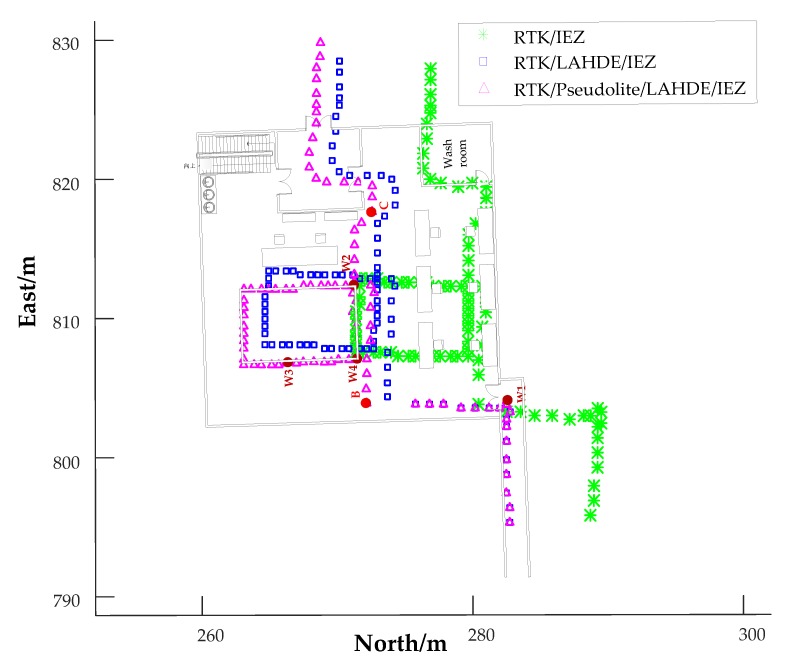
The trajectories of indoor corridor and indoor room parts ignoring positioning error at point A of Person A.

For better presenting the effectiveness of LAHDE algorithm, we ignored positioning errors of point W2, and then placed the pedestrian trajectories at the indoor map, as shown in Figure 14. The location error of the RTK/IEZ method without using LAHDE was 7.616 m, while the location error of the RTK/LAHDE/IEZ method was 0.876 m, an 88.5% reduction in positioning errors.

From Table 3, we can see that the proposed integrated solution of both Person A and Person B can achieve high-precision positioning, although the positioning errors of IEZ algorithm of Person B was lower than that of Person A.

### 5.2. The Performance of the LSMBCPD-IEZ Algorithm

**Figure 15:**
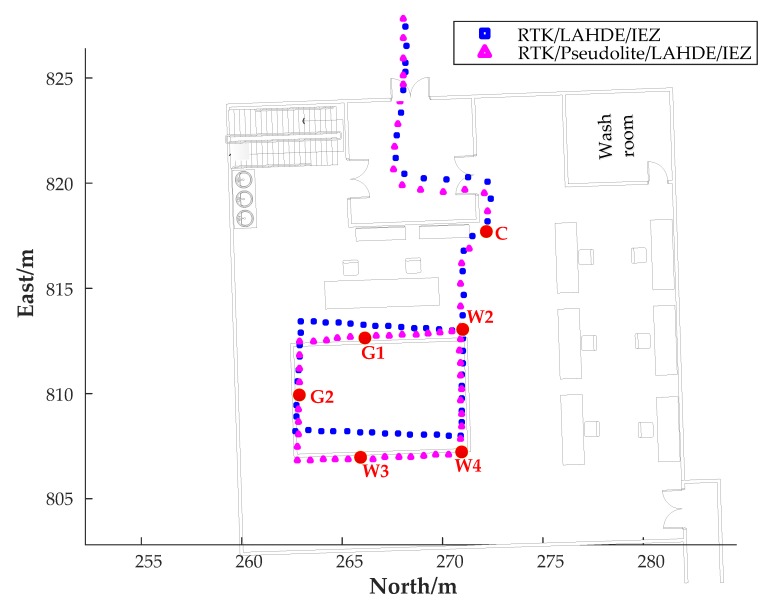
The trajectories of indoor corridor and indoor room parts ignoring positioning error at point B.

To evaluate the local search method based on carrier phase difference with the assistance of IEZ, we ignored the location error of the point B. The trajectories of RTK/Pseudolite/LAHDE/IEZ integrated solution and the RTK/LAHDE/IEZ integrated solution were placed on the map of the room, as shown in Figure 15, and the location error is shown in Table 4. As can be seen, the proposed LSMBCPD-IEZ was effective in eliminating the accumulative error of the IMU-based pedestrian navigation system. The average positioning error was less than 0.4 m. The location error of point C was larger than other waypoints because of the wall disturbances.

**Figure 16:**
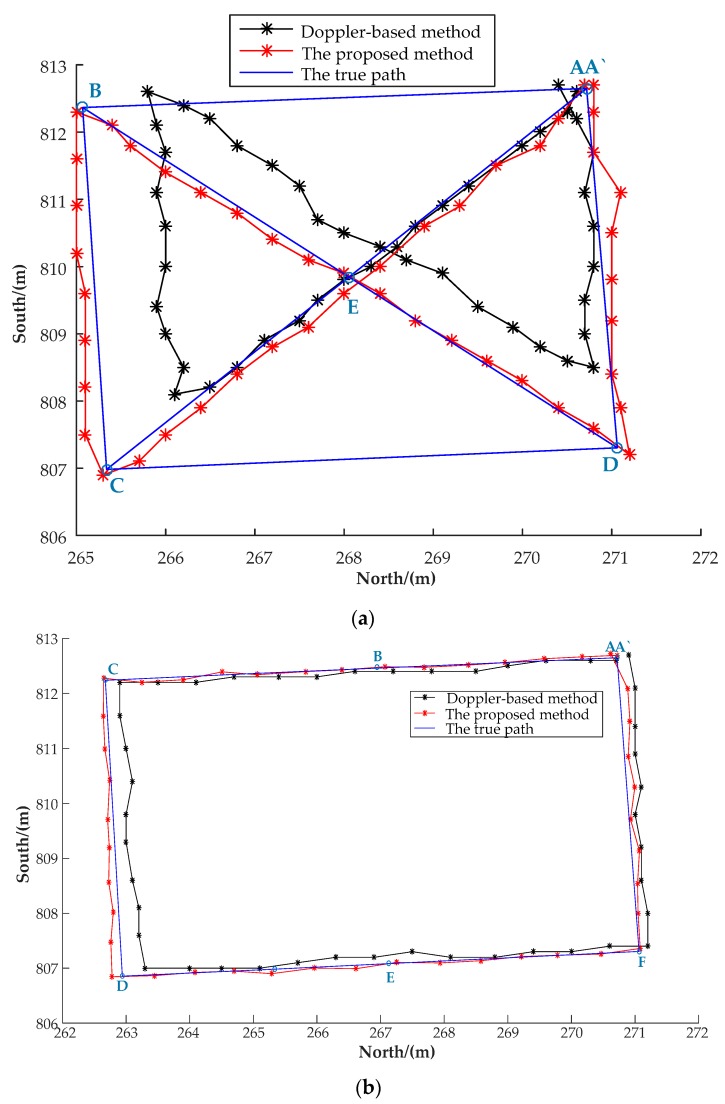
Evaluation of algorithms. (**a**) The trajectories of Person A. (**b**) The trajectories of Person B.

To verify the superiority of the proposed LSMBCPD-IEZ algorithm over the recently published pseudosatellite Doppler-based positioning algorithm, two experiments were carried out in the room by Person A and Person B, as shown in Figure 16 along two routes, respectively. One route was A-C-B-D-A` (A) and the other route was A-B-C-D-E-A` (A). The location errors are shown in Table 5 and Table 6, respectively. As can be seen, the proposed LSMBCPD-IEZ had higher positioning accuracy than the Doppler-based positioning method and the positioning accuracy of the proposed LSMBCPD-IEZ of Person B was almost as high as that of Person A, which shows that the performance of the proposed LSMBCPD-IEZ algorithm was hardly affected by different people.

**Figure 17:**
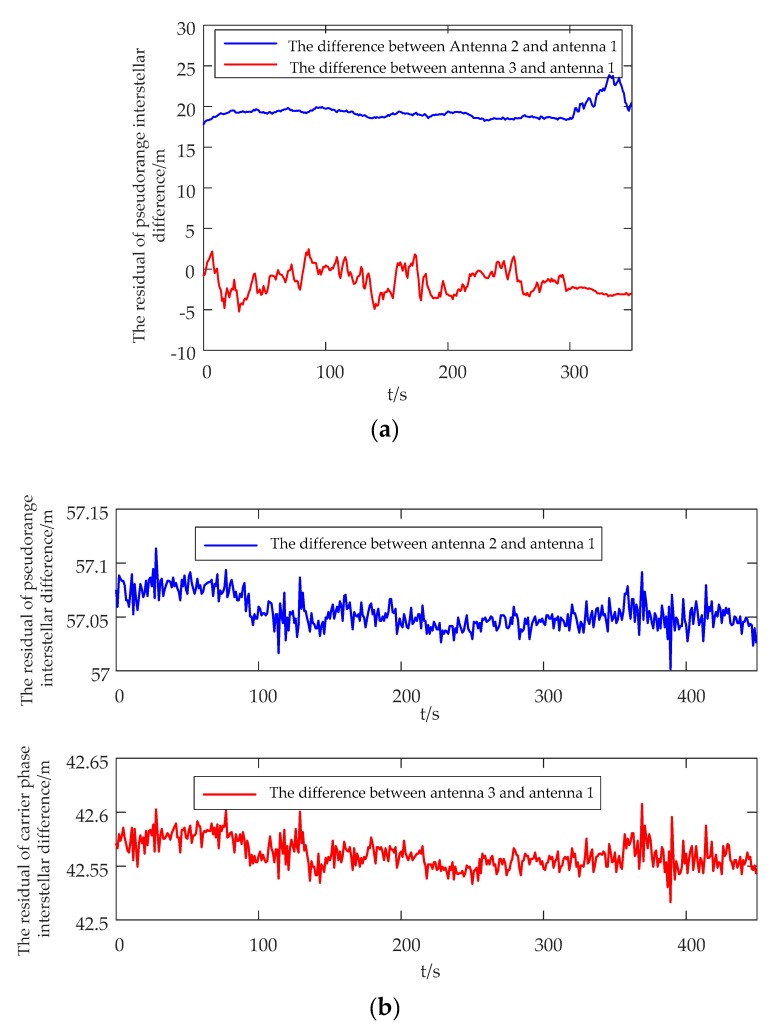
The residual of interstellar difference. (**a**) The residual of pseudorange interstellar difference. (**b**) The residual of carrier phase interstellar difference.

Figure 17 shows the residual of interstellar difference in indoor environments. It can be seen that, compared with pseudorange, the change of carrier phase was less affected by the indoor environment and had a stronger resistance to multipath.

## 6. Conclusions and Future Work

In this paper, a RTK/Pseudolite/LAHDE/IMU-PDR integrated pedestrian navigation system for cooperation scenes including urban, indoor corridors, and indoor room was proposed. A test verification system was developed to collect and process the data from RTK, pseudolite and IMU-PDR, and manmade landmarks to record the positions, and then filter algorithms were developed using MATLAB to fuse the recorded data using RTK/IMU-PDR, RTK/LAHDE/IMU-PDR, and RTK/Pseudolite/LAHDE/IMU-PDR, respectively. Although the proposed system seemed to be difficult to implement in real conditions because the amount of instrumentation used was high and cumbersome, it can be replaced by one smartphone and a pair of shoes in the future. RTK, pseudolite, and manmade landmarks were used as aiding systems to alternatively provide corrections to IMU-PDR in different environments because they work in complementary environments and the IMU-PDR can maintain the positioning continuity of system when the three absolute positioning methods were not available in the scene switching areas and wooded area. Pseudolite was deployed in indoor rooms to provide satellite-like navigation signals, which can be supported by the commercial receiver chip. A local search method based on carrier phase difference with the assistance of IEZ was proposed to achieve high-precision in indoor rooms positioning. Experimental results showed that, although the performance of the IEZ algorithm differed between different people, the IMU-PDR drift error can be reduced effectively during the whole trajectory, except for the scene switching areas, by the proposed system. The proposed local search method based on carrier phase difference with the assistance of an IEZ algorithm can achieve higher accuracy which was hardly affected by different people.

In the future, our work will focus on the studies of landmark detection methods and the scene recognition methods to make the proposed pedestrian navigation system smarter without using human behavior. We also will study pedestrian navigation technology for stairs and elevators.

## Figures and Tables

**Table 1:** Quality indicators.

Indicators	Quality
1	Location is unavailable or invalid
2	Single point positioning
3	Pseudorange differential positioning
4	RTK fixed solution
5	RTK floating point solution
6	Inertial navigation positioning
7	Fixed Position

**Table 2:** Comparison of location errors of Person A.

Location Errors (m)	A	W1	B	W2	W3	W4	C
IEZ	9.78	14.87	14.90	15.40	14.66	14.72	15.16
RTK/IEZ	2.47	8.38	8.73	9.35	8.66	8.69	9.10
RTK/LAHDE/IEZ	1.77	2.45	2.21	2.70	1.89	2.11	2.39
RTK/Pseudolite/LAHDE/IEZ	1.77	2.45	0.27	0.31	0.22	0.35	0.67

**Table 3:** Comparison of location errors of Person A and Person B.

Location Errors (m) of Person A	A	W1	B	W2	W3	W4	C
RTK/Pseudolite/LAHDE/IEZ	1.77	2.45	0.27	0.31	0.22	0.35	0.67
RTK/Pseudolite/LAHDE/IEZ	1.06	2.03	0.22	0.27	0.36	0.29	0.81

**Table 4:** Comparison of location errors of Person A.

Location Error(m)	W2	G1	G2	W3	W4	C
RTK/LAHDE/IEZ	0.31	0.90	0.95	0.97	0.61	1.03
RTK/Pseudolite/LAHDE/IEZ	0.31	0.10	0.42	0.22	0.35	0.67

**Table 5:** Comparison of location errors of Person A.

Location Error(m) of Person B	A	C	B	D	A`
The proposed method	0.10	0.08	0.14	0.10	0.13
Doppler-based method	0.13	1.36	0.73	0.64	0.32

**Table 6:** Comparison of location errors of Person B.

Location Error(m) of Person A	A	B	C	D	E	F	A`
The proposed method	0.10	0.13	0.14	0.173	0.21	0.11	0.15
Doppler-based method	0.10	0.19	0.21	0.41	0.32	0.145	0.20

## References

[1] Borenstein J., Feng L. Gyrodometry: A new method for combining data from gyros, and odometry in mobile robots. Proceedings of the IEEE International Conference on Robot Automation.

[2] Foxlin E. (2005). Pedestrian Tracking with Shoe-mounted Inertial Sensors. IEEE Comput. Graph. Appl..

[3] Nilsson J.O., Skog I., Händel P. Performance characterisation of foot-mounted ZUPT-aided INSs and other related systems. Proceedings of the International Conference on Indoor Positioning and Indoor Navigation.

[4] Fan Q., Zhang H., Sun Y., Zhu Y., Zhuang X., Jia J., Zhang P. (2018). An Optimal Enhanced Kalman Filter for a ZUPT-Aided Pedestrian Positioning Coupling Model. Sensors.

[5] Rajagopal S. (2008). Personal Dead Reckoning System with Shoe Mounted Inertial Sensors. Master’s Thesis.

[6] Borenstein J., Ojeda L. (2009). Heuristic Reduction of Gyro Drift in Gyro-Based Vehicle Tracking. Int. J. Veh. Inf. Commun. Syst..

[7] Jiménez A.R., Seco F., Prieto J.C., Guevara J. Indoor pedestrian navigation using an INS/EKF framework for yaw drift reduction and a foot-mounted IMU. Proceedings of the Workshop on Positioning Navigation & Communication.

[8] Jiménez A.R., Seco F., Zampella F., Prieto J.C., Guevara J. Improved heuristic drift elimination (iHDE) for pedestrian navigation in complex buildings. Proceedings of the International Conference on Indoor Positioning and Indoor Navigation.

[9] Li T., Zhang H., Gao Z., Chen Q., Niu X. (2018). High-Accuracy Positioning in Urban Environments Using Single-Frequency Multi-GNSS RTK/MEMS-IMU Integration. Remote Sens..

[10] Niu Z., Nie P., Tao L., Sun J., Zhu B. (2019). RTK with the Assistance of an IMU-Based Pedestrian Navigation Algorithm for Smartphones. Sensors.

[11] Bi J., Wang Y., Li X., Qi H., Cao H., Xu S. (2018). An Adaptive Weighted KNN Positioning Method Based on Omnidirectional Fingerprint Database and Twice Affinity Propagation Clustering. Sensors.

[12] Jung S., Lee C.O., Han D. Wi-Fi fingerprint-based approaches following log-distance path loss model for indoor positioning. Proceedings of the Mtt-s International Microwave Workshop Series on Intelligent Radio for Future Personal Terminals.

[13] Devanshi Agrawal S., Singh S. (2014). Indoor Localization based on Bluetooth Technology: A Brief Review. Int. J. Comput. Appl..

[14] Naya F., Noma H., Ohmura R., Kogure K. Bluetooth-based Indoor Proximity Sensing for Nursing Context Awareness. Proceedings of the Wearable Computers.

[15] Ni L.M., Liu Y., Lau Y.C., Patil A.P. (2004). LANDMARC: Indoor Location Sensing Using Active RFID. Wirel. Netw..

[16] Ozdenizci B., Ok K., Coskun V., Aydin M.N. Development of an Indoor Navigation System Using NFC Technology. Proceedings of the Information and Computing (ICIC), 2011 Fourth International Conference.

[17] Tiemann J., Eckermann F., Wietfeld C. Multi-user interference and wireless clock synchronization in TDOA-based UWB localization. Proceedings of the International Conference on Indoor Positioning & Indoor Navigation.

[18] Pannuto P. Ultra-wideband and indoor localization. Proceedings of the Workshop on Hot Topics in Wireless.

[19] Holm S. Ultrasound positioning based on time-of-flight and signal strength. Proceedings of the 2012 International Conference on Indoor Positioning and Indoor Navigation (IPIN).

[20] Ruiz A.R.J., Granja F.S., Honorato J.C.P., Rosas J.I.G. (2012). Accurate Pedestrian Indoor Navigation by Tightly Coupling Foot-Mounted IMU and RFID Measurements. IEEE Trans. Instrum. Meas..

[21] Ferreira A.G., Fernandes D., Catarino A.P., Rocha A.M., Monteiro J.L. (2019). A Loose-Coupled Fusion of Inertial and UWB Assisted by a Decision-Making Algorithm for Localization of Emergency Responders. Electronics.

[22] Xu Y., Chen X., Cheng J., Zhao Q., Wang Y. Improving tightly-coupled model for indoor pedestrian navigation using foot-mounted IMU and UWB measurements. Proceedings of the Conference: 2016 IEEE International Instrumentation and Measurement Technology Conference (I2MTC 2016).

[23] Zhuang Y., Lan H., Li Y., El-Sheimy N. (2015). PDR/INS/WiFi Integration Based on Handheld Devices for Indoor Pedestrian Navigation. Micromachines.

[24] Huang G., Ji Y.F., Sun X.Y. (2016). Design on Pseudolite Signal TransmitterBased on GPS Navigation System. Radio Eng..

[25] Fujii K., Sakamoto Y., Wang W., Arie H., Schmitz A., Sugano S. (2015). Hyperbolic Positioning with Antenna Arrays and Multi-Channel Pseudolite for Indoor Localization. Sensors.

[26] Huang L., Gan X., Yu B., Zhang H., Li S., Cheng J., Liang X., Wang B. (2019). An Innovation Fingerprint Location Algorithm for Indoor Positioning Based on Array Psedolite. Sensors.

[27] Gan X., Yu B., Huang L., Jia R., Zhang H., Sheng C., Fan G., Wang B. (2019). Doppler Differential Positioning Technology Using the BDS/GPS Indoor Array Pseudolite System. Sensors.

[28] http://www.techweb.com.cn/tele/2018-11-08/2711349.shtml.

[29] https://cn.amazfit.com/antelope.html.

[30] Ma M., Song Q., Gu Y., Li Y., Zhou Z. (2018). An Adaptive Zero Velocity Detection Algorithm Based on Multi-Sensor Fusion for a Pedestrian Navigation System. Sensors.

[31] Zhang R., Yang H., Höflinger F., Reindl L.M. (2017). Adaptive Zero Velocity Update Based on Velocity Classification for Pedestrian Tracking. IEEE Sens. J..

[32] Ojeda L., Borenstein J. (2007). Non-GPS navigation for security personnel and first responders. J. Navigat..

[33] Zhu R., Wang Y., Yu B., Gan X., Jia H., Wang B. (2020). Enhanced heuristic drift elimination with adaptive zero-velocity detection and heading correction algorithms for pedestrian navigation. Sensors.

[34] http://www.unicorecomm.com/files/PDF/Ch/HPL/UB482_UserManual_Ch%20V2.0.pdf.

[35] http://file.yizimg.com/516728/2019106-91831645.pdf.

[36] Abdulrahim K., Hide C., Moore T., Hill C. Aiding MEMS IMU with building heading for indoor pedestrian navigation. Proceedings of the 2010 Ubiquitous Positioning Indoor Navigation and Location Based Service.

